# Hydrophobicity, rather than secondary structure, is essential for the SRP dependent targeting of GPR35 to the ER membrane

**DOI:** 10.1007/s10863-019-9785-0

**Published:** 2019-01-31

**Authors:** Jon K. Cherry, Cheryl A. Woolhead

**Affiliations:** 1 Institute of Molecular, Cell and Systems Biology, College of Medical, Veterinary and Life Sciences, University of Glasgow, Glasgow, G12 8QQ UK; 2 Present Address: The Centre for Bacterial Cell Biology, Medical School, Newcastle University, Newcastle upon Tyne, NE2 4AX UK

**Keywords:** Signal recognition particle, Endoplasmic reticulum, Membrane protein, Protein folding

## Abstract

The folding and targeting of hydrophobic transmembrane domains poses a major challenge to the cell. Several membrane proteins have been shown to gain some degree of secondary structure within the ribosome tunnel and to retain this conformation throughout maturation. However, there is little information on one of the largest classes of eukaryotic membrane proteins; the G protein-coupled receptors (GPCRs). In this study we show that the signal anchor domain of GPR35 remains in an extended conformation whilst exiting the ribosome tunnel, the polypeptide chain then forms interactions with components of the SRP targeting pathway, and the Sec61 translocon, resulting in a compacted conformation prior to integration into the ER membrane. We conclude that transmembrane structure is most likely adopted after the domain leaves the ribosome tunnel and that the interaction of the signal anchor with SRP is dependent on the native levels of hydrophobicity within the first transmembrane domain. Therefore, we propose a mechanism by which the first transmembrane domains of multi-spanning membrane proteins adopt compacted structures following SRP targeting but before insertion into the ER membrane.

**Electronic supplementary material:**

The online version of this article (10.1007/s10863-019-9785-0) contains supplementary material, which is available to authorized users.

## Introduction

The co-translational targeting of integral membrane proteins (IMPs) poses a major challenge to the cell, as they must remain insertion competent while their highly hydrophobic transmembrane domains are transferred from the ribosome, through the aqueous cytosol and into the lipid bilayer, via the Se61 machinery (SecYEG in bacteria). In the last decade, increasing amounts of research has suggested that the biogenesis of integral TM domains may begin far in advance of reaching the translocon.

The ribosome tunnel itself has been highlighted as one of the major sites for the generation of both secondary structure, in the form of α-helical domains, and tertiary structure, such as small hairpin–like domains, in membrane proteins (Holtkamp et al. 2015). The size of the tunnel from the peptidyl transferase centre (PTC) to the point of exit is ~100 Å in length, and its diameter ranges from 10 Å at its narrowest point to 20 Å at the vestibule (Ban et al. 2000; Bhushan et al. 2010; Voss et al. 2006). It has been shown to be both structurally and biochemically diverse, housing around 30 amino acids in an extended conformation or up to 65 amino acids in a compacted helical conformation (Lu and Deutsch 2005a; Ziv et al. 2005). The wall of the tunnel is lined with ribosomal RNA and ribosomal proteins (uL4, uL22 and uL23), which have been identified to interact with the nascent chain in a sensory and regulatory manner, often influencing processes such as biogenesis, targeting and membrane insertion. Several studies in the last decade have provided evidence of ribosomal proteins not only line, but protrude into the tunnel, generating distinct zones of helix stabilization that could play a key role in promoting peptide folding (Bhushan et al. 2010; Lu and Deutsch 2005b, 2008; Woolhead et al. 2004).

As folded domains of TM proteins make their way to the exit site of the tunnel, the external surface of the ribosome becomes primed for an interaction with the signal recognition particle (SRP). During the initial stages of SRP mediated targeting, the ribosomal protein L17 in eukaryotes and the tunnel loop domain of L23 in the prokaryotic ribosome were proven to interact specifically with hydrophobic membrane segments. Biochemical and biophysical assays have identified that these proteins play a key role in sensing the nascent chain, and subsequently initiate downstream events that stabilise structure that may already be present in the nascent polypeptide chain (Robinson et al. 2012; Woolhead et al. 2004).

The interactions within the tunnel of the ribosome are believed to initiate SRP recruitment to the external surface, enabling docking on the globular domain of uL23. In eukaryotes, SRP is made up of a large 7S RNA and 6 proteins including SRP54 that are essential for the capture and protection of the emerging TM domain. Upon exiting the ribosome, the targeting component of the nascent chain is subsequently housed in the M domain of SRP. Structural analysis of the M domain has found that it is made up of four helices ordered around a central hydrophobic core, which is believed to accommodate ~10 residues of mainly α-helical structure (Hainzl et al. 2011; Janda et al. 2010; Keenan et al. 1998). This would suggest that nascent chains choosing to interact with SRP might be required to take up an α-helical conformation either before or upon binding the hydrophobic groove, in line with previous reports of signal peptides and anchors forming structure before release from the ribosome.

Further structural studies have provided us with insights into how SRP, after binding the nascent peptide, targets to the ribosome-nascent chain complex to the Sec61 translocon (Kobayashi et al. 2018; Lee et al. 2018; Gao et al. 2017). The Sec translocon, which resides in the lipid membrane as a heterotrimeic complex, is made up of α, β and γ-subunits. High resolution structures of the both prokaryotic and eukaryotic Sec translocon have enabled us to deduce that the translocation of proteins across the membrane occurs through a narrow pore within the complex (Voorhees et al. 2014). As the ribosome engages the Sec translocon, a structural change occurs, allowing the open translocon to laterally move the nascent chain into the lipid bilayer of the ER (Plath et al. 1998; Sadlish et al. 2005; Van Den Berg et al. 2004; Voorhees and Hegde 2016). The lateral movement of membrane proteins has been well studied using both single spanning and polytopic membrane proteins, in which obtaining the correct orientation and secondary structure before integration is essential, in addition to SRP targeting the translocon pore may aid this process (Hessa et al. 2005). Secondary structure formation within the Sec translocon is poorly understood, but there is evidence that the environment provided by the pore could enable TM domains to sample multiple conformations (Goder and Spiess 2003). Interactions between the translocating nascent chain and the α-subunits of the channel, as well as accessory factors such as TRAM may impact on the ability of the peptide chain to fold in this environment (Heinrich et al. 2000; McCormick et al. 2003; Sadlish et al. 2005). As secondary structure formation is deemed necessary prior to integration, it is a realistic prospect that folding in some domains may occur during translocation by the Sec machinery.

In this study, we investigate the biogenesis of the signal anchor domain of the GPCR; GPR35. A class A GPCR, this orphan receptor will be used to report where the signal anchor begins to form secondary structure. Using biochemical techniques in both prokaryotic and eukaryotic translation systems, we analyse secondary structure in the first TM domain as it makes its way through the ribosome tunnel and investigate any evolutionary differences that may exist between different ribosome species. We show that the signal anchor domain of GPR35 is unfolded in the upper and mid regions of the tunnel, as well as in the lower regions where it first encounters SRP. We show that the signal anchor of GPR35 is capable of binding SRP, an interaction driven by hydrophobicity within the domain. Altering the hydrophobicity within the first TM segment destabilizes the interaction with SRP and subsequently reduces insertion of GPR35 into the ER membrane. Finally, secondary structure is measured during the insertion of the first TM domain into the Sec61 translocon. This provides us with evidence that the signal anchor is in an α-helical conformation and the second TM domain, which resides in the ribosome tunnel, remains extended.

## Results

### GPR35 signal anchor domain is extended in the ribosome tunnel

**Fig. 1:**
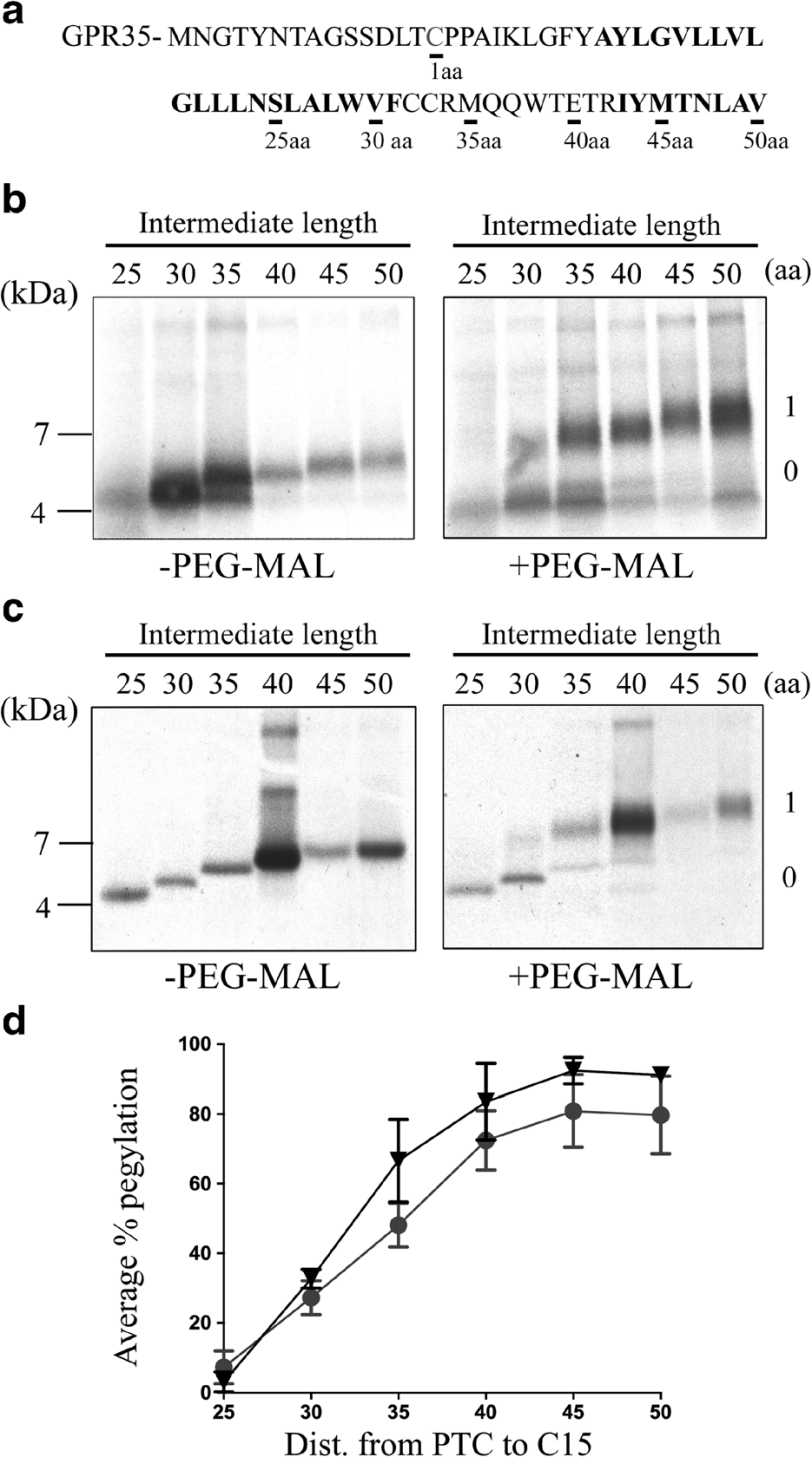
**TM1 of GPR35 exists in an extended conformation in the ribosome tunnel.** (**a**) The first 65 residues of human GPR35 are shown, with the position of the single cysteine (C15) residue, required for pegylation, labelled as amino acid 1. TM domains 1 and 2 are highlighted using boldface. Intermediate lengths of GPR35, used for in vitro pegylation experiments, are underlined and the length is denoted below in amino acids (aa). Autoradiographs of radiolabelled translation products generated from stalled intermediates of between 25 and 50 amino acids in length were expressed in the (**b**) prokaryotic S-30 coupled transcription/translation system and the (**c**) eukaryotic Wheat Germ (WG) translation system. Intermediate length was measured from the peptidyl-transferase centre (PTC) to the marker cysteine (C15). Translation reactions were split into two, with one half being incubated with 1 mM PEG-MAL (+PEG-MAL) and the other half incubated in buffer as a control (-PEG-MAL). A representative gel displays how the non-pegylated (0) and pegylated (1) samples were resolved by SDS-PAGE (15% Tricine). A gel-shift of ~ 5 kDa occurs if the translation product was successfully pegylated. (**d**) Quantification of the total pegylation of individual intermediates was carried out for both the S-30 transcription/translation system (●) and the WG translation system (▼). The y-axis shows percentage of intermediates successfully pegylated and was obtained by pixel densitometry (Image-J) and calculated using [pegylated band/ (unpegylated band + pegylated band)]. The average percentage pegylation is calculated from an *n* = 3 replicates, error bars are + SD

Previous studies analysing the formation of secondary structure in both signal peptides and integral membrane domains, have identified zones within the ribosome tunnel where conformational changes in the nascent peptide are likely to occur. Conditions within both the prokaryotic and eukaryotic tunnel have proven themselves favourable for generation of structured nascent peptides such as α-helices and tertiary hairpins (Thommen et al. 2017). To determine whether such structural formation is likely to occur in the signal anchor domain of a model GPCR; GPR35, a study was set up to investigate where, if at all, the first TM domain is likely to form a structured polypeptide. To measure compaction within a ribosome-bound peptide, a well-practiced pegylation assay was carried out. The success of this assay relies on the accessibility of a molecule of PEG-MAL, to a specifically placed cysteine residue (the marker cysteine (MC)) within the nascent polypeptide and the known dimensions of the ribosome tunnel. The bulky polyethelene glycol tails of PEG-MAL prevent the molecule entering far into the ribosome tunnel. Therefore, pegylation of the MC only occurs out with the tunnel, hence providing us with an indication of peptide compaction. To explore the formation of secondary structure within the signal anchor domain of GPR35, ribosome nascent chain complexes (RNCs) were synthesized in vitro, with individual intermediates generated from linear DNA lacking a stop codon. Intermediates of various lengths, ranging from 25-50aa from the peptidyl transferase centre (PTC) to the MC were generated, increasing at intervals of 5aa within the range (Fig. 1a).

This set of experiments were carried out in the in vitro S-30 prokaryotic transcription/translation system (Fig. 1b). At an intermediate length of 25aa, no pegylation took place. At this point in the translation process, an intermediate of this length would be expected to be buried within the ribosome tunnel, and hence should be inaccessible to the PEG-MAL molecule regardless of the conformation within the first TM domain. As the nascent chain increased in length to 30aa, the MC advanced 5aa closer to the exit site. In doing so, a small fraction of the translation product becomes pegylated resulting in a shift in size of the translation product (Fig. 1b). As the distance between the PTC and MC increased further, between 35-40aa, we see greater levels of pegylation within the translation product. Further lengths of 45aa and 50aa shows maximal levels of pegylation (approximately 80%), suggesting the marker cysteine had fully exited the ribosome tunnel at that point and becomes fully exposed. Collectively, these results indicate that that an unstructured nascent is present in the ribosome tunnel. To see if the lack of secondary structure within the GPR35 signal anchor was not an artefact of the prokaryotic ribosome, a similar set of experiments were carried out in the eukaryotic wheat germ (WG) translation system. As expected, the results remained consistent in the eukaryotic system (Fig. 1c, d). Pegylation of intermediates at 30aa from the PTC indicated that the TM domain of GPR35 was unstructured in the upper and middle regions of the ribosome tunnel. Near identical levels of pegylation, also indicates little variation in the role played by the respective ribosome tunnels in contributing to secondary structure within the nascent chain (Fig. 1d).

To validate the pegylation data gathered on GPR35, two subsequent membrane proteins were tested as controls; Bacterioopsin (Bop) (Ortenberg and Mevarech 2000) and subunit c of the F_0_ component of the ATP synthase (F_0_c) (Van Der Laan et al. 2004) (Fig. S1A and S2A). Bop was used due to its structural resemblance to a GPCR. It provided us with a seven TM protein that was expressed natively in a prokaryotic system, yet could be compared structurally to the eukaryotic, GPR35. The first TM domain of Bop was discovered to have a folding profile, which was remarkably similar to that of GPR35 (Fig. S1B, C, D). Although, neither the 25aa nor the 30aa intermediate could be expressed in the S-30 system, the levels of pegylation in the 35-50aa peptides followed an identical pattern to that data gathered with GPR35. A full range of intermediates could be expressed in the WG expression system and they showed consistent levels of pegylation to GPR35. This suggested that the first TM domain of Bop traversed the ribosome in an unstructured state, eluding to a possible trend in seven TM domain proteins. The F_0_c protein, previously shown by Robinson et al. (2012) to compact in the ribosome tunnel, showed a very different pegylation profile. Ribosome nascent chain complexes (RNCs) were synthesized in the S-30 expression system only, with intermediates ranging in size from 35-70aa, taking into account the likelihood that compaction would occur. At 35aa from the PTC, the MC remained unpegylated unlike the same sized intermediates in GPR35 and Bop. The first indication of pegylation of F_0_c was when the MC was approximately 45aa from the PTC suggesting compaction of the nascent chain was occurring. Following this, further intermediates became maximally pegylated (approximately 80%) maintaining a similar trend to the previous assays.

The pegylation assays led to the conclusion that GPR35 exists in an extended conformation and shows no sign of forming secondary structure, in the ribosome tunnel. This result compares well with previous studies that found extended peptides (3.4 Å per aa) would require approximately 30aa to traverse the ribosome tunnel, where as a fully compacted peptide would require approximately 65aa (1.5 Å/aa) to span the same distance (Lu and Deutsch 2005a). If compaction of the signal anchor did occur the MC would be labelled approximately 45aa from the PTC and not before, with this data therefore ruling out the existence of significant secondary structure within the exit tunnel.

### GPR35 signal anchor reaches SRP as an extended nascent chain

To explore further the conformational changes occurring in the nascent chain of GPR35 as it becomes exposed at the exit site of the ribosome, and subsequently undergoes the process of membrane targeting, an assay was set up to utilize the prospective interaction with SRP. Using the chemical cross-linker bis(sulfosuccinimidyl)suberate (BS^3^), we attempted to monitor interactions between the nascent chain and components of the SRP targeting pathway, and imply from these the conformational changes occurring in the signal anchor domain as it encounters SRP.

**Fig. 2:**
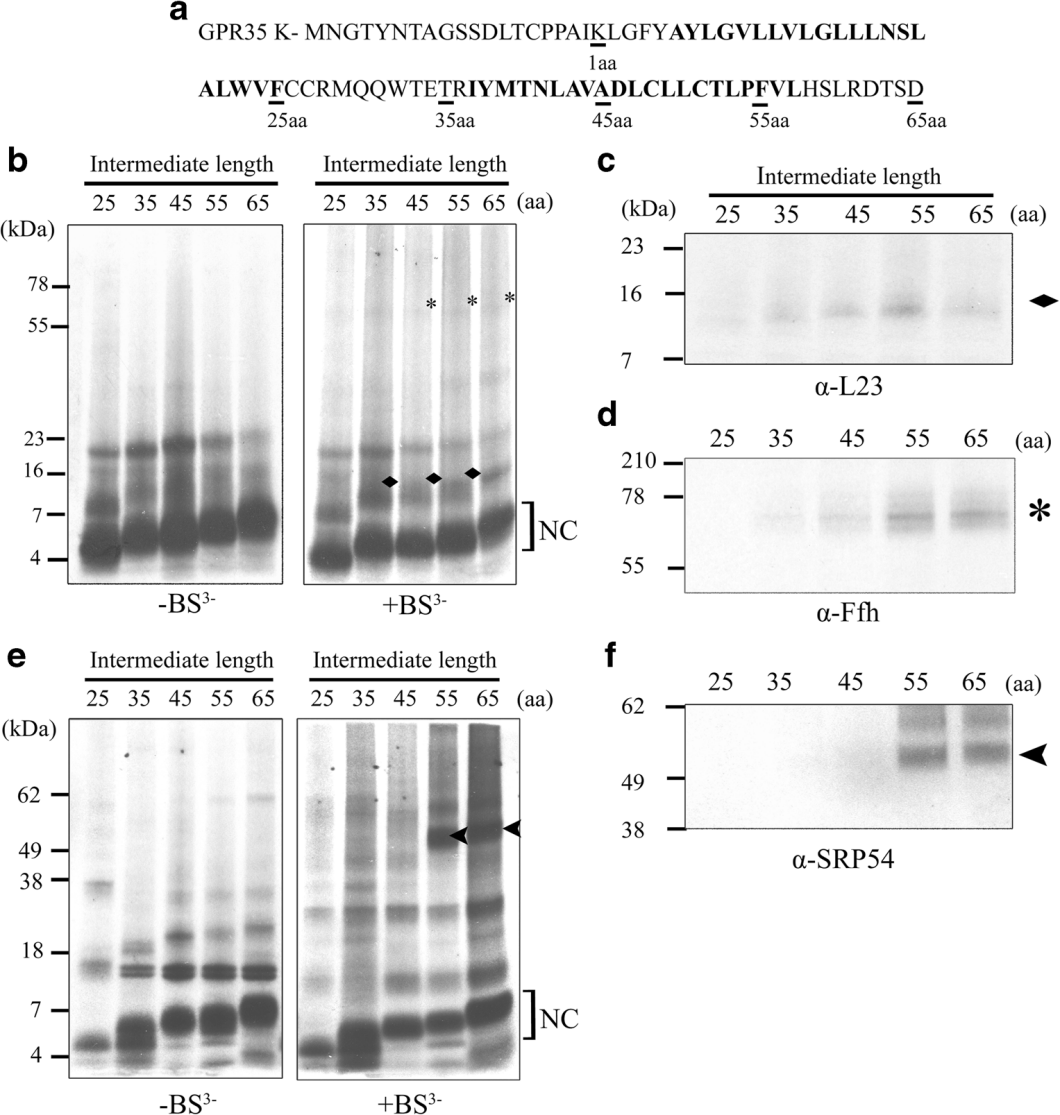
**TM1 of GPR35 binds SRP upon exiting the ribosome ET.** (**a**) The first 85 residues of human GPR35 are shown with the position of the single lysine (K20), required for cross-linking with BS^3^, labelled as amino acid 1. TM domains 1 and 2 are highlighted using boldface. Intermediate lengths of GPR35, used for in vitro cross-linking experiments, are underlined and the length is denoted below in amino acids (aa). Autoradiographs of radiolabelled translation products generated from stalled intermediates of between 25 and 65aa in length can been seen before (-BS^3^) and after (+BS^3^) treatment with BS^3^, when resolved on a 15% tricine gel. Intermediate length was measured from the PTC to K20. (**b**) Intermediate lengths of GPR35 were expressed in the S-30 transcription/translation system. Prominent cross-links displaying the correct molecular weight for GPR35-L23 and GPR35-Ffh complexes have been marked by a  and * respectively. Products of the cross-linking assay in the prokaryotic system were subjected to immunoprecipitation with (**c**) anti-uL23 serum, (**d**) anti-Ffh serum, showing specific cross-links were occurring between these proteins and the ribosome bound nascent chain. (**e**) Similar sized intermediate lengths of GPR35 were expressed in the eukaryotic Rabbit Reticulocyte Lysate (RRL) system. Cross-links displaying the correct molecular weight for GPR35-SRP54 are marked by a ◄. (**f**) Products of the cross-linking assay in the eukaryotic system were immunoisolated with an anti-SRP54 serum, which displayed specific cross-links to a number of stalled intermediates

A single lysine residue (K20), found 5aa upstream of the signal anchor, makes the GPR35 WT construct suitable for this cross-linking assay as it has a native marker lysine (MK) for interacting with BS^3^. Initial experiments were performed in the prokaryotic S-30 in vitro transcription/translation expression system, as antibodies for the detection of SRP targeting components uL23 and Ffh (bacterial SRP54) were available. As the bacterial and eukaryotic SRP pathways share common ancestry, we were confident that interactions between the GPR35 nascent polypeptide and the components of the prokaryotic system take place. Radiolabelled intermediates ranged from 25-65aa (from the PTC to K20), at increasing at intervals of 10aa. This range allowed us to explore the interactions taking place both during and after TM1 has traversed the ribosome tunnel. In the absence of the cross-linker molecule BS^3^, the translated nascent peptides can be seen to increase in size as they increase in length from 25-65aa (Fig. 2b, e). Weaker background bands, believed to result from endogenous DNA/RNA are also visible (Fig. 2b, e). In the presence of BS^3^, the different sized peptides can once again be detected; however, there is also evidence of interactions occurring between the nascent chain and components of the SRP pathway. The first evidence of this is when the MK is 35aa from the PTC, placing it extremely close to the exit site of the ribosome tunnel and suggesting that the nascent chain is likely to be in an extended conformation. This band can be immunoprecipitated with an antibody raised to the uL23 protein and is the first sign of cross-linking to a protein involved in the targeting of GPR35 (Fig. 2c). The cross-links between the nascent chain and uL23 also exist at intermediates of 45aa and 55aa in length, suggesting the nascent chain may reside in the distal regions of the tunnel for an extended period. Also between the lengths of 45-55aa, a higher cross-link appears at approximately 55 kDa and can again be detected in the 65aa sample (Fig. 2b). These larger cross-links can be immunoprecipitated by the antibodies raised to the Ffh protein (Fig. 2d). The results seem to show a sequential interaction for the nascent chain passing from the uL23 to Ffh protein, with cross-links weakening in uL23 and strengthening in Ffh after approximately 55aa.

We performed simultaneous cross-linking experiments in a eukaryotic system to test the timing of an interaction between GPR35 and eukaryotic SRP. Using the Rabbit Reticulocyte Lysate (RRL) expression system, intermediates of the same length and range were generated (Fig. 2e), producing results similar to those seen in the prokaryotic system. In the absence of BS^3^, translation product representative of each intermediate could be detected. Also, present in each sample at approximately 16 kDa was a double band representative of the protein haem (found in all RRL samples isolated by centrifugation through a sucrose cushion) and an unknown band at ~28 kDa. In the presence of BS^3^, cross-links between the nascent chain at lengths 55 and 65aa could be detected with a protein of approximately 50 kDa (Fig. 2e). The bands that appeared in the cross-linked samples were immunoprecipitated with an antibody raised to SRP54, indicating an interaction was taking place between the nascent chain and eukaryotic SRP (Fig. 2f). Cross-linking to SRP54 at 55aa is in agreement with the results seen in the prokaryotic system, suggesting the GPR35 signal anchor encounters SRP as an extended peptide. Prolonged cross-linking of GPR35 between uL23 and SRP may also suggest the first signs of compaction in TM1; however, this cannot be certain at this point.

### Hydrophobicity, and not secondary structure, within the signal anchor drives an interaction with SRP

It has been suggested that both ∝−helical structure and hydrophobicity play a key role in initiating an interaction between the nascent chain and SRP at the exit tunnel of the ribosome (Schibich et al. 2016). A number of nascent polypeptides have shown the ability to form ∝ − helical structures upon exiting the ribosome, and SRP can house an ∝−helix of approximately 10aa in its hydrophobic groove. However, based on our pegylation and crosslinking assays, this does not seem to be the case for the first TM domain of GPR35. Previous results show an extended polypeptide in the exit tunnel; therefore leading us to hypothesise that its affinity for SRP was solely driven by hydrophobicity.

**Fig. 3:**
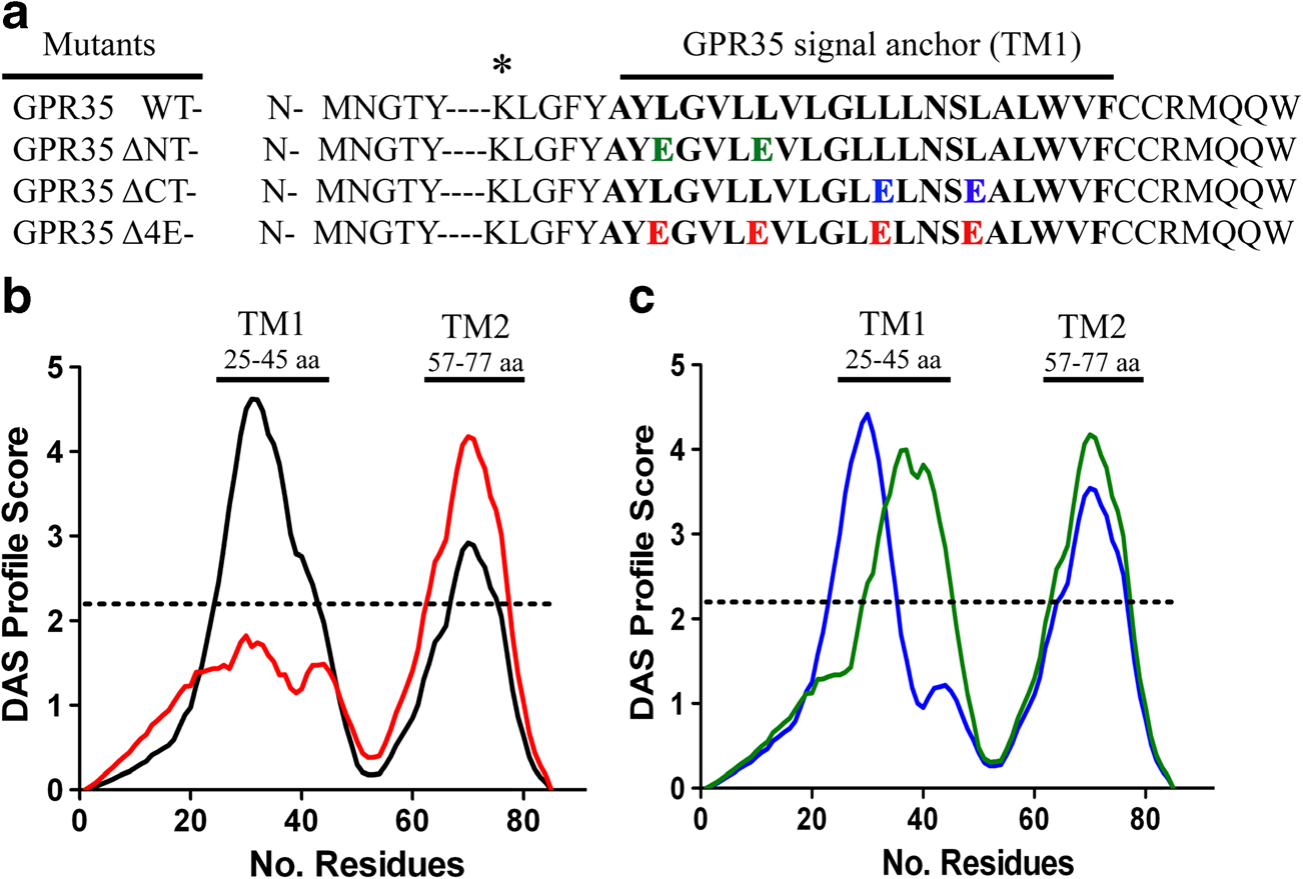
**Substitution of Leucine for Glutamic acid residues severely reduces the hydrophobicity in transmembrane domain 1 of GPR35. **(**a**) Substituted Leucine to Glutamic acid residues are highlighted in green when at the N-terminus of TM domain 1, blue when in the C-terminus and red when all four exist throughout. The marker lysine (K20) is marked with by a *. (**b**) The hydrophobicity of GPR35 WT (black) and GPR35 Δ4E (red) was calculated using the Dense Alignment Surface (DAS) method (Cserzö et al. 1997). Four Glutamic acid substitutions at positions 27, 31, 36 and 40 within GPR35 TM domain 1 (red), reduce the hydrophobicity below the strict cut-off point (dotted line) set out by the DAS method indicating the presence of a TM domain. (**c**) Substitutions of Leucine to Glutamic acid at residues 27 and 31 (green), and residues 36 and 40 (blue) reduce the hydrophobicity in the N-terminus and C-terminus of GPR35 TM domain 1 respectively

To investigate the effect a loss in hydrophobicity within the signal anchor had on SRP binding, a similar cross-linking experiment was once again undertaken. Three constructs were generated to significantly reduce hydrophobicity in the N-terminus (ΔNT), C-terminus (ΔCT) or entire signal anchor domain (Δ4E) (Fig. 3a). Substitution of leucine to glutamic acid residues were introduced in order to have the most dramatic effect, without altering the entire sequence of the first TM domain. Substitutions resulting in the Δ4E variant abolished the hydrophobicity within the signal anchor (Fig. 3c); however, the ΔNT or ΔCT variants retained enough hydrophobicity to be recognised as a TM protein on the DAS model.

**Fig. 4:**
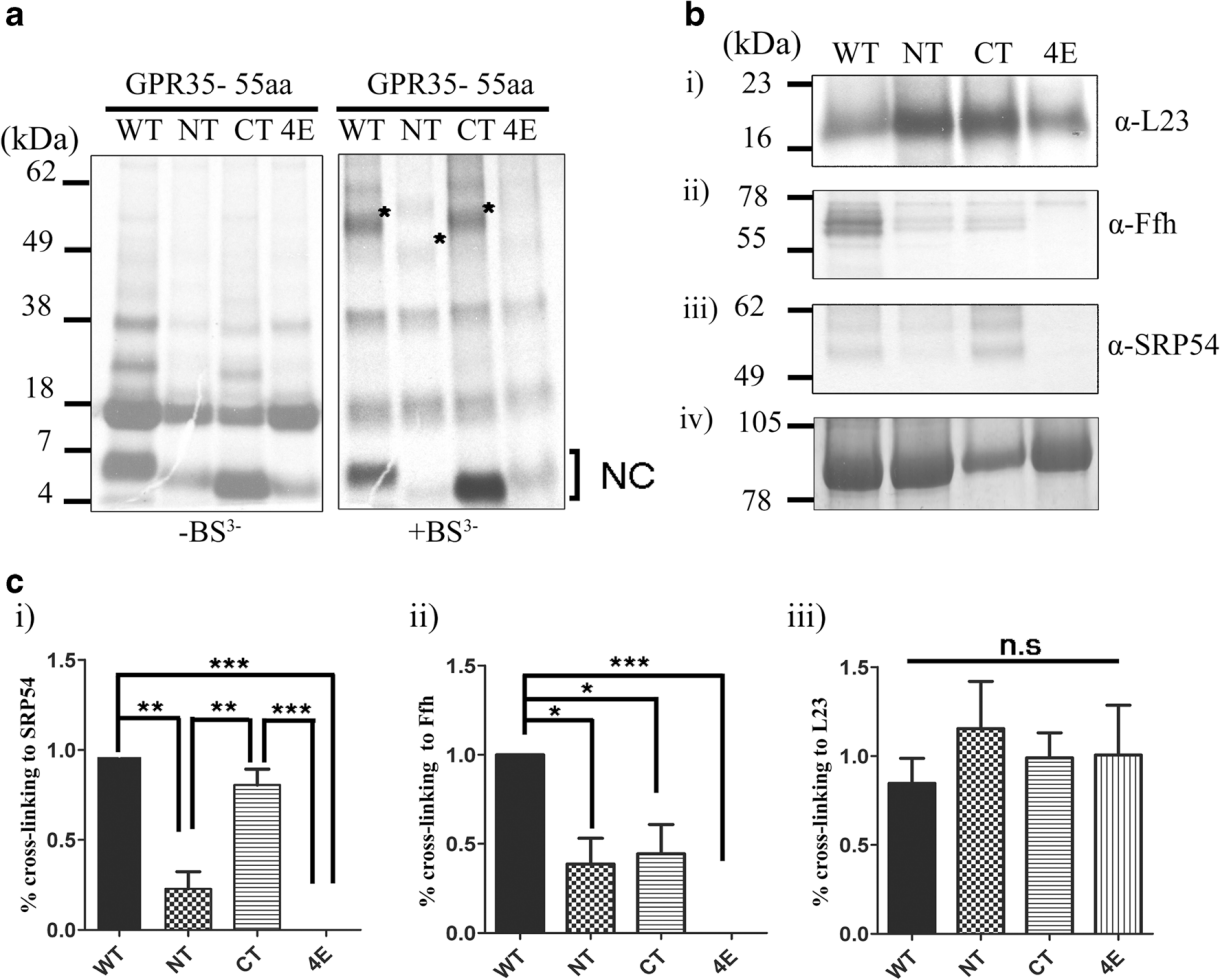
**Cross-linking assay of GPR35 hydrophobic mutants reduce binding to prokaryotic and eukaryotic SRP. **(**a**) Autoradiographs of radiolabelled translation products of GPR35 hydrophobic mutants, at a length of 55 amino acids, can been seen before (-BS^3^) and after (+BS^3^) treatment with BS^3^ when resolved on a 15% SDS-PAGE gel. Prominent cross-links displaying the correct molecular weight for GPR35-SRP54 complexes have been marked with a *. (**b**) Cross-linking samples were immunoisolated with, **(i)** anti-SRP54 serum, **(ii)** anti-Ffh serum or **(iii)** anti-L23 serum. SDS-PAGE gels used for autoradiographs were rehydrated and stained with Coomassie Blue, which provided a loading control for quantification of cross-linking **(iv)**. (**c**) Bar graphs show the average percentage cross-linking to **(i)** SRP54 **(ii)** Ffh, or **(iii)** L23 for individual intermediates. Immunoprecipitation bands were quantified using Image J software. All percentage cross-linking values were calculated using [Immunoprecipitation product/Coomassie loading control] and adjusted for background. Average percentage cross-linking is calculated from an *n* = 3 replicates. Error bars are ± SD groups were compared using one-way ANOVA with Tukey’s post hoc comparisons. *p < 0.05, **p < 0.005, ***p < 0.001

Cross-linking with BS^3^ was then carried out using the 55aa intermediate (K20 is 55aa from the PTC), as this peptide length showed cross-linking to uL23, Ffh and SRP54. As before, the uL23 protein was used as a cross-linking control in the S-30 expression system and was shown to interact in the same manner with all four intermediates following immunoprecipitation (Fig. 4b i). However, differences can be noticed between the WT and the 3 variants when cross-linked with both eukaryotic and prokaryotic SRP. In the prokaryotic S-30 system, both the ΔNT and ΔCT intermediates form a much weaker interaction with Ffh (Fig. 4b ii) and were found to interact with a lower than 50% affinity compared to the WT GPR35. Although this is the case, both intermediates are capable of rescuing the loss of Ffh binding seen with the Δ4E intermediate (Fig. 4b ii, c ii). When the same experiment was carried out in the eukaryotic RRL system, a different effect could be seen when comparing the four intermediates. The WT intermediate was once again shown to have the strongest interaction to SRP whereas the Δ4E intermediate, containing the four leucine to glutamic acid mutations, could not be cross-linked to SRP54 at all (Fig. 4b iii). However, a noticeable difference was detected between the binding of SRP54 to the ΔNT and ΔCT intermediates (Fig. 4b iii). The ΔNT intermediate interacts with SRP54 in a manner that is more representative of the Δ4E intermediate, barely rescuing cross-linking to SRP at all. The cross-linking efficiency between the nascent chain and SRP54 is reduced to approximately 20% (Fig. 4c iii), a much greater effect than the one that occurred between the same construct and Ffh. The opposite effect occurs between the ΔCT intermediate and SRP54, with SRP binding approximately 75% of that seen with the WT construct (Fig. 4b iii, c iii). The results highlight a possible difference between prokaryotic and eukaryotic SRP cross-linking, potentially alluding to a different mechanism of recognition or simply a higher level of complexity behind SRP binding in eukaryotes.

### Postranslational targeting and integration of GPR35

**Fig. 5:**
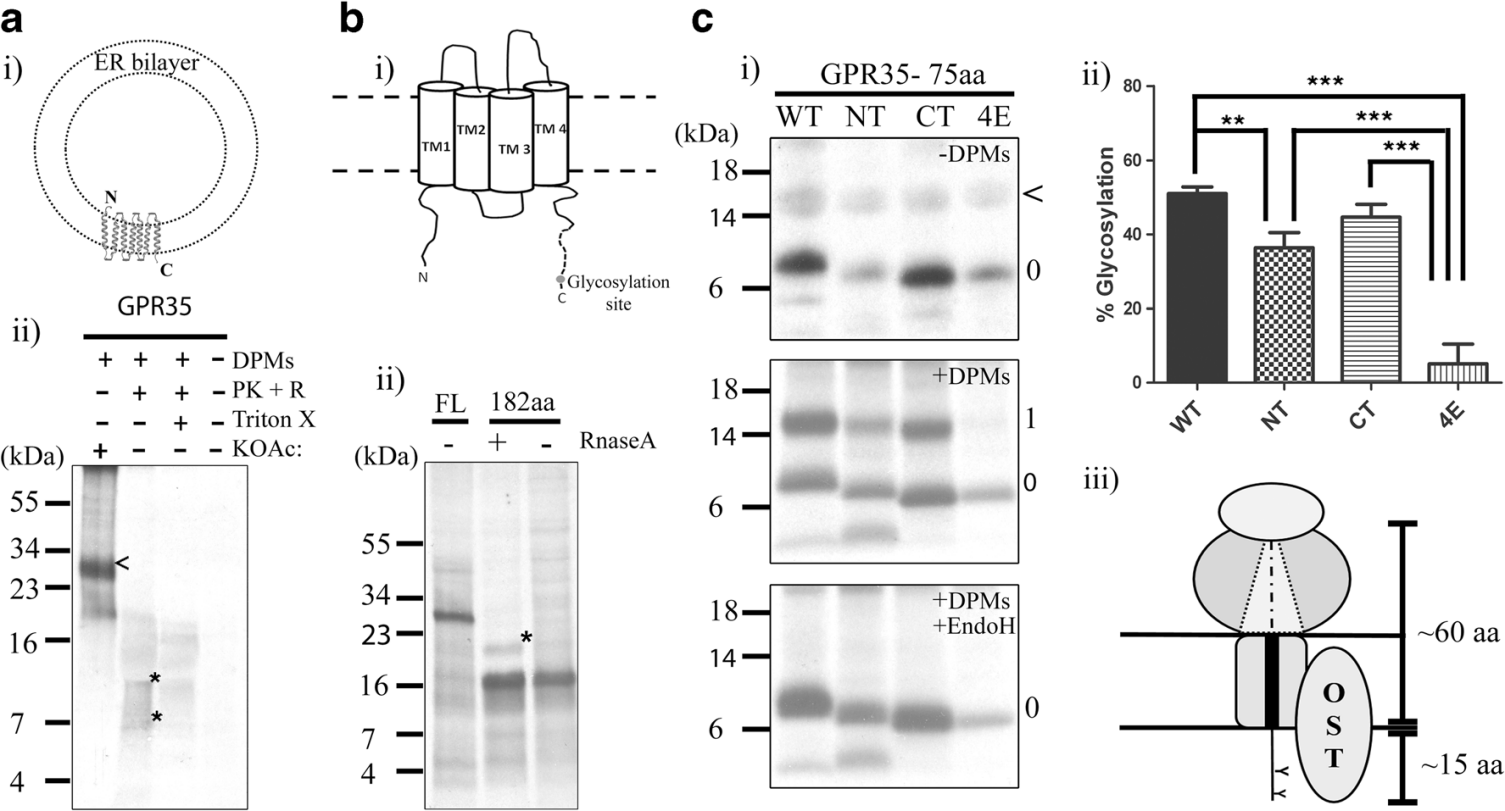
**Digestion and glycosylation assays indicate hydrophobicity of the transmembrane domain 1 in GPR35 is required for membrane insertion. (a) (i)** A schematic diagram indicating how GPR35 is correctly orientated in the ER membrane**.** The seven transmembrane domain protein is inserted N-terminally, so that the N-terminus it is exposed to the endoplasmic reticulum (ER) lumen. **(ii)** Radiolabelled full-length (FL) GPR35 was targeted to dog pancreas microsomes (DPMs) that were added to the RRL translation mix. DPMs were washed with KOAc to ensure GPR35 was fully inserted and not just associated with the lipid bilayer. Digestion assays used proteinase K (PK) and RnaseA (R), assessed if GPR35 was successfully inserted into DPMs. Successful insertion of FL GPR35 should provide protection of TM domains and luminal loop regions. The addition of the detergent Triton X-100 (Triton X) will permeabilize the DPM bilayer enabling digestion of entire GPR35 protein. GPR35 cannot be isolated in samples lacking DPMs. **(b) (i)** Schematic diagram shows how a GPR35 intermediate of 182aa length should be orientated in the DPM bilayer. A synthetic glycosylation site introduced at the C-terminus should determine orientation. **(ii)** A glycosylation assay to assess if the GPR35 182aa intermediate is correctly orientated in the membrane. Selective release and glycosylation of the 182aa intermediate by RNaseA, shows GPR35 is orientated correctly in DPMs**.** The glycoslyated translation product is indicated by a *. **(c) (i)** Glycosylation assay to assess translocation of the GPR35 TM1 domain hydrophobicity mutants. Ribosomes displaying radiolabelled N-terminal intermediates of GPR35 at lengths of 75 amino acids. Translation product and glycoslyated translation product are indicated by 0 and 1 respectively, with the presence of the background haem band marked with a <. Products were resolved on a 15% Tricine gel. **(ii)** Glycosylation results were quantified using Image J software. All percentage glycosylation values were calculated using [glycosylated product/ total translation product] and adjusted for background. Average percentage of glycosylation is calculated from an *n* = 3. Error bars indicate SD and groups were compared using one-way ANOVA with Tukey’s post hoc comparisons. *p < 0.05, **p < 0.005, ***p < 0.001. **(iii)** Model showing the approximate extension length required to span the ribosome-Sec complex, as estimated from the glycosylation data, with the presence of an α-helical TM domain within the Sec channel

Due to the interaction detected between GPR35 and SRP54, GPR35 was expected to become integrated into the ER membrane via the Sec61 translocon. To ensure successful integration and correct orientation, digestion and glycosylation experiments were set up. Radiolabelled GPR35 nascent chains were generated using the RRL in vitro translation system, to which dog pancreas microsomes (DPMs) were added to provide an ER membrane component and the end point for GPR35 insertion. The initial experiment to test the success of GPR35 insertion into the ER membrane was carried out using a digestion assay. This relied on the protease activity of proteinase K (PK) to assess whether or not integrated domains of mature GPR35 had gained protection from the membrane bilayer (Fig. 5a i). In the presence of DPMs, the translated full length GPR35 can be detected at approximately 30 kDa, indicating correct insertion (Fig. 5a ii). In the absence of DPMs no full-length GPR35 was detected, indicating that GPR35 only isolates with the membrane. The insertion of mature GPR35 was confirmed upon the addition of PK at which point the 30 kDa band disappeared and was replaced by bands at ~7 kDa. These bands represent TM fragments of the GPR35 that have been protected from PK degradation by the lipid bilayer. Insertion of GPR35 into the membrane was further confirmed by disrupting the permeability of the bilayer with detergent (Fig. 5a ii). The bands present in the sample containing DPMs could no longer be detected because disruption of the membrane allows for the degradation of the full GPR35 protein. Although the digestion assay confirms integration into the membrane, at no point was N-linked glycosylation of full length GPR35 detected. A native glycosylation site on the GPCR N-terminus should interact with the oligosaccharyl-transferase (OST) complex close to the Sec61 translocon upon integration, providing information on whether GPR35 has taken up the correct orientation. Therefore, the results indicate that GPR35 can be successfully inserted into DPMs but the orientation of the full-length protein is unknown.

To further investigate the orientation of GPR35 post insertion, a glycosylation assay was designed. On this occasion an engineered glycosylation site was placed at the C-terminal end of an 182aa intermediate of GPR35 (Fig. 5b i). This glycosylation site was incorporated into the DNA template through PCR, at the sequence encoding for extracellular loop 2 (positioned on the luminal side of the ER of the mature GPR35) extending the protein by 18aa. This extension placed the glycosylation site 16aa downstream from TM4 and theoretically in range of the OST complex (Fig. 5b i). The template lacks a stop codon, generating stalled ribosome bound nascent chains that are only released by the addition of RNaseA and EDTA to the translation mix. This selective release from the ribosome enables the comparison between the glycosylated and unglycosylated peptides, to confirm that GPR35 is inserted in the correct orientation.

The results of this assay show convincingly that GPR35 is capable of inserting into DPMs with the correct orientation (Fig. 5b ii). Upon translation and isolation of the membrane integrated 182aa intermediate, a single band could be detected at ~16 kDa representing an unglycosylated ribosome bound peptide. Upon addition of RNaseA and EDTA to the translation mix, the peptidyl-tRNA bond was broken and the ribosomal subunits removed, allowing for the release of the GPR35 C-terminus. Subsequent translocation of the C-terminus produced two bands when analysed by gel electrophoresis, one representing the 182aa intermediate and a second higher band representative of the glycosylated product. This suggests that GPR35 is in the correct orientation, therefore allowing us to carry out further structural evaluations on its signal anchor domain as it becomes inserted into the membrane.

Collectively these results suggest that in this system GPR35 is capable of making its way successfully to the membrane bilayer, where it correctly orientates. This integration process is heavily linked to the native hydrophobicity within the first TM domain, which aids the co-translational targeting process carried out by SRP.

### Hydrophobicity in the signal anchor of GPR35 plays a key role in translocation

Previous results suggest that hydrophobicity within the signal anchor domain plays a key role in targeting GPR35 to the Sec61 translocon. We now would like to address whether altering the hydrophobicity within the signal anchor affects the translocation and integration processes. To determine this, we once again used the constructs ΔNT, ΔCT and Δ4E, and quantify how they integrate into the membrane of DPMs. To do this, we generated radiolabelled intermediates (75aa in length) long enough to interact with the OST found adjacent to the Sec61 translocon and assessed N-linked glycosylation at the native site at the N-terminus of GPR35 (Fig. 5c iii). In the absence of DPMs, one band representative of the translation band exists (Fig. 5c i). Upon the addition of DPMs to the RRL translation reaction, the GPR35 WT intermediate, as expected, becomes targeted and successfully inserted. Approximately half the translation product shows a shift in MW from ~7 to ~14 kDa, representative of glycosylation at the N-terminal glycosylation site. To ensure the higher MW band was glycosylated translation product, the DPMs were incubated with the enzyme endoglycosidase H **(**Endo H), which is capable of removing sugar group generated from a glycosylation event. In the presence of Endo H, the higher MW bands associated with glycosylation were successfully removed (Fig. 5c i). A similar assay was carried out using the ΔNT and ΔCT constructs, where both intermediates showed a reduction in translocated product in comparison to the WT GPR35 intermediate, which may have been representative of a loss of interaction between the nascent chain and SRP, as seen in the cross-linking results (Fig. 4b, c). Generally, the effect on translocation in the ΔNT intermediate was significantly greater when compared to the WT intermediate, than what was seen in the ΔCT intermediate (Fig. 5c ii). Finally, the Δ4E intermediate showed extremely low levels of the glycosylation product being produced when compared to each of the other intermediates (Fig. 5c i, c ii). This would suggest that the N-terminus of the Δ4E intermediate was unsuccessfully translocated across the ER membrane due to of a loss in hydrophobicity, leading to poor SRP targeting or poor insertion into the DPM membrane.

This result highlights the importance of hydrophobicity not only for an interaction with SRP, but for downstream events such as translocon interactions and lipid bilayer integration. Remaining hydrophobicity within the ΔNT and ΔCT intermediates, although significantly reduced when compared to the WT intermediate, enables translocation and integration into the ER membrane. Reducing the hydrophobicity below the level of a recognisable TM domain all but abolishes correct integration by Sec61.

### Assessing the secondary structure of GRP35 transmembrane domain 1 during translocation

**Fig. 6:**
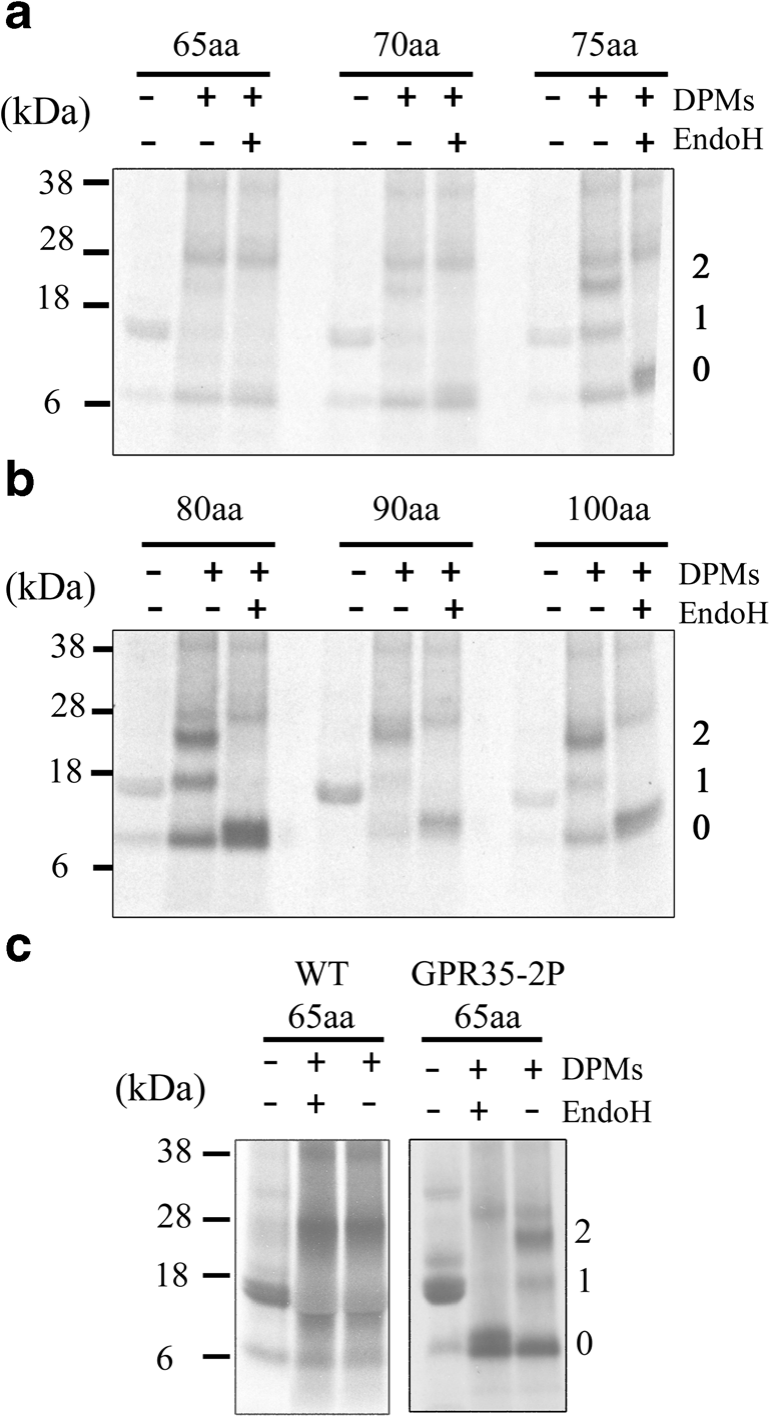
**Analysis of GPR35 glycosylation to detect the formation of secondary structure in the Sec61 translocon.** Autoradiographs of radiolabelled translation products of GPR35-gly construct. Intermediate lengths ranging from 65-75aa (**a**) and 80-100aa (**b**) of GPR35 were expressed in an in vitro RRL translation reaction. Reactions were split into three, with one third being incubated without DPMs, another being incubated in the presence of DPMs and the final third being incubated in the presence of DPMs and Endo H. Autoradiographs indicate the presence of an unglycosylated translation product (0), a singly glycosylated translation product (1) and a doubly glycosylated product (2). In addition, the presence of a background band at approximately 16 kDa, which is representative of haem, is indicated by a <. (**c**) Two leucine to proline substitutions at positions 34 and 40 were introduced into the first TM domain of the GPR35-gly construct, generating GPR35-2P (2P). The presence of two prolines act as a helix breaker and generating an extended intermediate, which acted as a negative control for glycosylation. Glycosylation of the 65aa GPR35-2P confirms the presence of a helix in TM domain 1

Finally, as we suspect that the signal anchor domain of GPR35 remains in an extended conformation during membrane targeting by SRP, it is expected that the α-helical structure must form before release from the translocon. Therefore, we expect a conformational change within the GPR35 signal anchor during insertion into the Sec61 translocon. To assess this conformational change, we can use a similar assay used by (Whitley et al. 1996), where the length of the glycosylated nascent chain indicates the presence of secondary structure. In an extended conformation, with each residue contributing ~3.4 Å to the length of the nascent chain, roughly 65aa should be required to span the distance from the PTC to the OST. The result of folding within the signal anchor, whilst inside the Sec61 translocon, will increase the required peptide length to 75aa (Fig. 5c iii). For this experiment a construct with a second glycosylation site was designed to avoid interference with a previously described background band, believed to be haem, which runs at the same size as some of the smaller glycosylated intermediates. In the absence of DPMs from the RRL translation system, a band correlating to the size of each unglycosylated intermediate could be detected. Also present was the background haem band (~16 kDa) seen previously arise upon isolation of RNCs without DPMs present. Upon the addition of DPMs, the appearance of two bands of increased molecular weight (MW) could be detected along with the unglycosylated intermediate (Fig. 6a). The two bands of increased MW were the result of a single or double glycosylation event taking place at the N-terminus of GPR35 intermediates. The 65aa intermediate shows no sign of glycosylation at either glycosylation site, suggesting that it is not long enough at this point to interact with the OST complex. Although the first glycosylation site within this intermediate is 70aa from the PTC and theoretically should be capable of low levels of glycosylation, there is none to be detected. This could indicate that this site in particular does not become glycosylated efficiently or may not be recognised by the OST due to its close proximity to the start codon. In the 70aa intermediate however, signs of weak glycosylation can be detected at both sites, suggesting that ~70 residues are required for glycosylation to occur. Glycosylation then appears to occur at increased levels in the 75aa intermediate, suggesting it is at an optimum length for interaction with the OST; this level of glycosylation was maintained throughout the longer peptide lengths (Fig. 6b). To ensure the two higher MW bands were a result of glycosylation, the isolated DPMs were treated with the enzyme EndoH. Upon addition of the enzyme, the existence of the two higher MW bands disappear, hence confirming that they result from glycosylation of the native intermediate (Fig. 6a and b). Collectively, these results indicate that a folding event has taken place in the signal anchor domain, during point of interaction with SRP and insertion into the Sec61 translocon.

The introduction of two proline residues at positions 31 and 40 within the TM1 domain of GPR35, further strengthened the argument for the formation of secondary structure within the translocon (Fig. 6c). The 65aa intermediate, previously incapable of becoming glycosylated, showed evidence of two glycosylation bands when proline residues were introduced into the first TM domain of GPR35. This suggests that the TM domain is unstructured and remains in an extended conformation, hence interacting with the OST machinery.

Glycosylation as a marker for the presence of secondary structure in translocating intermediates of GPR35 was capable of determining that the N-terminus and specifically the first TM domain forms a compacted structure in the Sec61 translocon. The lack of glycosylation products at 65aa from the PTC and the presence of glycosylated intermediates at 70aa, and increasingly at 75aa, indicate the presence of a helix-like domain during translocation. At this point we are unable to confirm whether it is due to an interaction with SRP or the translocation events that begins folding of TM1. In either case, it is an essential event in the biogenesis of GPR35, preparing it for integration into the membrane.

## Discussion

Transmembrane domain biogenesis has been well studied in the last decade, yet precise data on where and why such an event takes place is poorly understood. Many IMPs are known to be inserted into their respective membranes co-translationally and for this to occur correctly, insertion competency must be maintained. Several research groups have highlighted key steps between translation and integration where an IMP can begin to form secondary and even tertiary structure. In this study, we have assessed the conformational changes occurring in the signal anchor domain of a GPCR, GPR35, during the early stages of membrane protein biogenesis. GPCRs are one of the largest and most widely studied family of membrane proteins; however, little research has focused on the folding of their TM domains prior to membrane insertion. Our work indicates that unlike many IMPs studied to date, the first TM domain of GPR35 exists in an extended state until entry into the Sec translocon. In vitro pegylation assays were the first to indicate that the nascent polypeptide, containing the GPR35 signal anchor, could traverse and exit the tunnel in a relaxed conformation. Cross-linking data confirmed the lack of secondary structure within the first TM domain, as nascent chain lengths of between 35aa and 45aa could interact with ribosomal protein uL23 and SRP/Ffh respectively. Interestingly, the lack of secondary structure in the signal anchor had no ill effects on the interaction or co-translational targeting of the nascent polypeptide by SRP. Mutations in the TM domain, which decreased the hydrophobicity, resulted in a loss of interaction between the nascent chain and SRP, and thus affected integration of the signal anchor into the ER. This seemed to suggest that intrinsic hydrophobicity within the TM domain and not secondary structure was the driving force for entering the co-translational targeting pathway. Finally, a glycosylation assay was set up to assess the folding profile of the signal anchor upon entry into the Sec61 translocon. This is the first point at which the TM domain of the nascent GPCR polypeptide resembled an α-helical peptide.

Interestingly, the folding profile of this eukaryotic protein is unlike much of what has been observed previously with other TM domains or signal peptides whilst traversing the ribosome tunnel. To date a number of studies have shown TM domains have a high propensity for forming secondary and even tertiary structure before exiting the ribosome. However, here we present an integral domain of a eukaryotic membrane protein which remains extended until the point in which it is inserted into the Sec61 translocon. A number of studies have highlighted the role played by the ribosome tunnel in aiding the formation of secondary structure within TM segments of a translating nascent chain (Lu and Deutsch 2005a; Robinson et al. 2012; Woolhead et al. 2004). In particular, several studies have provided evidence for preferred ‘folding zones’ within the tunnel where compaction of the nascent chain takes place. A number of experiments suggest that the upper tunnel, near the PTC, is one such region where folding can occur. Ribosomal proteins uL4 and uL22, found in the mid-tunnel, are believed to either play a role in stabilizing a structure that had previously formed in the upper tunnel or even aid compaction within the nascent chain itself. Our results from both the pegylation and cross-linking assays suggest that compaction of GPR35 in the upper tunnel is highly unlikely, as nascent peptides can be both pegylated at ~30aa and cross-linked to uL23 as early as 25aa from the PTC. Cross-links identify the close proximity of the nascent chain to the ribosomal protein uL23, located at the base of the large subunit. It is therefore unlikely that a peptide of this length could become pegylated out with the tunnel, or interact with a protein at the exit port of the ribosome and contain secondary structure. However, in the last decade, it is the most distal regions of the tunnel where the greatest wealth of structural data within nascent peptides has appeared. This region has been the most frequently described ‘folding zone’ for secondary structure in TM domains of IMPs and the only region described for the formation of tertiary structure, such a β-hairpins or the complete formation of small proteins (Marino et al. 2016; Nilsson et al. 2015; Tu et al. 2014). In this study, we can track the TM domain as it moves through the tunnel and by varying the length of intermediates we can detect whether folding has taken place. Again the interaction with uL23 at intermediate lengths between 25-35aa suggests, even in the lower regions of the tunnel, compaction has not occurred. At this point the entire TM1 domain would be synthesised and shows little evidence of secondary structure formation during the early stages of synthesis. uL23 is known to play an important role in the targeting of membrane proteins, with its loop domain acting as a sensor in the recruitment of SRP.

As the peptide grows in length, an interaction with SRP can be identified. Again, the nascent chain appears to interact with SRP whilst in an extended conformation (cross-links both Ffh/SRP54 at ~45aa in length). At this intermediate length, for an interaction to be occurring between the nascent chain and SRP, the signal anchor would have traversed the tunnel and be located in the vestibule, with its N-terminus protruding from the exit port. Although, the GPR35 nascent chain meets with SRP in such an extended conformation, we are uncertain whether the entire TM domain remains so. Structural data analysing the interaction between a signal sequence and the M domain of SRP suggests 10 extended residues would be unlikely to fit in the hydrophobic groove (Janda et al. 2010). To date, most structural data suggests that the binding groove of SRP, which interacts directly with the nascent chain as it exits the ribosome, is likely to house an α-helical peptide of approximately 10aa in length (Halic et al. 2006; Janda et al. 2010; Keenan et al. 1998; Voorhees and Hegde 2015). Therefore, based on the structure of SRP, a compacted portion of N-terminus in the first TM of GPR35 would be more favourable. This being said, our study shows that it may be in fact hydrophobicity within the signal anchor that drives an SRP interaction, limiting structure to a minor role. By substituting 2 leucine for aspartic acid residues, at either the N-terminal or C-terminal end of the TM domain, the interaction between nascent chain and SRP was dramatically reduced. The introduction of all four leucine residues within the TM domain abolished the interaction completely. As a number of studies have previously suggested, hydrophobicity within a signal peptide or anchor domain is essential for an interaction with SRP and in the case of GPR35 it may be the driving force for initialising the co-translational targeting pathway. Furthermore, we observe an adverse effect on the integration of the signal peptide when the hydrophobicity is dramatically altered. Altering the hydrophobicity of the signal anchor domain results in a failure to initiate targeting to the ER, hence compromising the ability of the nascent polypeptide to become translocated and integrated into the membrane. Whether the loss of a hydrophobic TM segment impacts on the recognition of the nascent by ribosomal proteins or direct impacts the interaction with SRP it is unknown. However, sensing of a hydrophobic stretch in a nascent polypetide by proteins within the ribosomal tunnel has been shown to play a highly important role in the co-translational targeting pathway.

This work proposes that up until the point at which the first TM domain reaches the Sec61 translocon, the nascent GPR35 signal anchor remains extended or at best loosely folded. Although this is the case, ultimately it is vital that the signal anchor assumes an α-helical conformation before becoming integrated into the ER membrane. The translocon, through which the nascent polypeptide will pass, has been shown to provide a suitable environment for a compacted nascent chain, such as an α-helix. Indeed, a number of studies have shown that the Sec61 translocon is known to favour compacted or α-helical peptides (Hessa et al. 2005). Following a well-established method of studying helical domains in the Sec61 translocon, we see the GPR35 signal anchor domain change from an extended conformation to a compacted peptide. This suggests that the environment provided by the translocon is sufficient to generate a conformational change within the first TM domain, allowing it to be successfully integrated into the ER membrane. This work presents another example of the folding potential within a nascent peptide provided it is situated in the correct environment.

Helix formation within the TM domains of an IMP is a process that is fundamental for ensuring that the correct biogenesis of the protein is acquired. The ability for such proteins to obtain their structure as they progress through the co-translational targeting and integration cycle undoubtedly plays a role in deciding their fate. Our findings show that a TM domain, in a protein as integral as a GPCR, can remain in a relatively unfolded state until entry into the translocon. The ribosome tunnel has been increasingly thought of as an inducer and director of protein folding and targeting. However, the results shown here with regard to the signal anchor of GPR35 may suggest that there is a subset of membrane proteins that can remain unfolded until the point of membrane insertion.

## Materials and methods

### Plasmid construction

All plasmids used in this study are listed in Table S1. For transcription/translation experiments, intermediates of GPR35 were amplified from the construct pTrc99aGPR35 (pGPR35) for prokaryotic assays and the construct pcDNA3.1GPR35 (pcGPR35) for eukaryotic assays. For experiments using the *E.coli* S-30 extract system, GPR35 was placed under the control of a trc promoter and when using the Wheat Germ (WG) or Rabbit Reticulocyte Lysate (RRL) system, GPR35 was placed under the control of a T7 promoter. For pegylation experiments, a C8A mutation was carried out to remove a native cysteine residue. A marker cysteine (MC) residue, essential in the pegylation process, was introduced 10aa upstream of the signal anchor domain by a W15C mutation to yield pGPR35C (pTrc99a) and pcGPR35C (pcDNA3.1). Mutations that affected the properties of the first TM domain were incorporated into pGPR35 and pcGPR35; L27E, L31E, L34E and L40 resulted in pGPR35Δ4E; L27E and L31E resulted in pGPR35ΔNT; L34E and L40E resulted in pGPR35ΔCT. For experiments requiring glycosylation of pcGPR35, a single native glycosylation site was used or a second site was engineered by introducing an S residue between N6 and T7. This yielded the construct pcGPR35-gly. Mutations affecting secondary structure of the signal anchor domain were incorporated into pcGPR35-gly; L31P and L40P resulted in pGPR35-gly2P. Amplification reactions were carried out using an ExTaq PCR kit (TaKaRa), and site-directed mutagenesis was carried out using the QuikChange system (Stratagene).

### RNC preparation

S-30 extract was prepared from strain C41 essentially as described previously (Woolhead et al. 2006). Linear DNA was amplified from the appropriate constructs using an ExTaq PCR kit (TaKaRa). In these reactions, the 5′ primer was located upstream of either the trc promoter in pTrc99a (5′- CTGAAATGAGCTGTTGACAATTAATCATCCGG-3′) or the T7 promoter of pcDNA3.1 (5′-TAATACGACTCAC- TATAGGG-3′). The various 3′ reverse primers used, which are described in the Table S2, amplified internally from the GPR35 gene to produce DNA intermediates of the required length, lacking stop codons.

Purified amplified DNA was used in the S-30 coupled transcription/translation system; these reactions were performed in varying volumes principally as described previously for S-30 reactions. Briefly, a typical 50 μL reaction contained 1 μg DNA, 20 μL premix, 5 μL 1 mM L-amino acids (minus methionine), 15 μL S-30 extract, 20 μCi [35S] methionine, and an antisense oligonucleotide to SsrA at a concentration of 200 ng/mL. Reactions were incubated at 37 °C for 30 min and chilled on ice for 5 min.

For WG and RRL systems, in vitro transcription with T7 RNA polymerase was carried out on amplified DNA samples at 37 °C for 2 h. Purified RNA was used to generate [S^35^] methionine radiolabelled proteins in vitro; reactions were performed in varying volumes principally as specified by Promega Protocol and Application guide.

### Pegylation assays

As previously described (Lu and Deutsch 2005a), RNCs were pelleted through a sucrose cushion (100 μL; 0.5 M sucrose, 100 mM KCl, 5 mMMgCl2, 50 mM HEPES, 1 mM DTT (pH 7.5)) for 6 min at 436,000 g at 4 °C in a Beckman TLA-100 rotor. The pellet was resuspended in 30 μL of buffer (100 mM NaCl, 5 mM Mg2+, 20 mM HEPES, 50 mM DTT (pH 7.2)) by pipetting gently, avoiding the formation of bubbles. An equal volume of buffer containing 2 mM PEG-MAL was added (final PEG-MAL concentration was 1 mM) and incubated on ice for 2 h. The reaction was terminated by adding DDT (100 mM) and incubating at room temperature for 10 min. To precipitate the ribosome nascent chains, add 600 μL NaOAc (0.5 M (pH 4.7)) and 250 μL 2% cetyl trimethylammonium bromide (CTAB). The pellets were aspirated in 15 μL RNAaseA (1 mg/mL) to digest tRNA. Samples were heated in 2X sample buffer (4% SDS, 20% Glycerol, 0.12 M Tris pH 6.8, and 10% BME) at 95 °C for 5 min for analysis through Tricine SDS-PAGE.

### Chemical cross-linking and immunoprecipitation

Translation reactions (100 μL) were carried out as previously described to generate nascent peptides of desired lengths. A 7 μL portion of the reaction is overlayed onto a 50 μL sucrose cushion (tube A) while the remainder is overlayed onto a 100 μL sucrose cushion (tube B). RNCs were pelleted in a Beckman TLA-100 rotor for 6 min at 436,000 g at 4 °C to pellet the ribosome bound nascent chain. Pellet A was resuspended in 8 μL of RNC buffer, 100 μg/mL RNase A and 5 mM EDTA at incubated at 26 °C for 10 min. The sample was the heated in 2 X sample buffer at 95 °C for 5 min. Pellet B was resuspended in 88 μL of BS^3^ buffer (RNC buffer and 1 mM BS^3^) and incubated on ice for 2 h. The reactions are quenched on the addition of 5 ml of 1 M Tris pH 8.0 and incubated at 20 °C for 15 min. 7 μL of sample is added to 100 μg/ mL RNase A and 5 mM EDTA at incubated at 26 °C for 10 min and heated in 2 X sample buffer at 95 °C for 5 min. The remainder of the samples were TCA precipitated and used for immunoprecipitations with polyclonal rabbit antisera generated against the L23, Ffh and SRP54 peptides. All samples were resolved using Tricine or Tris-Bis SDS-PAGE gels.

### Insertion of GPR35 into dog pancreas microsomes

GPR35 intermediates produced through the eukaryotic RRL system were incubated in the presence of dog pancreas microsomes (DPMs) (80 equivalents/ml). The reactions were incubated at 30 °C for 30 min before ultracentrifugation for 30 min at 434,500 g, 4 °C to pellet the DPMs. The pellets were re-suspended in sample buffer and analysed through tricine SDS–PAGE and autoradiography. For digestion assays the pellets were washed with 3 M potassium acetate (KOAc) and repelleted before resuspension in 20 mM Hepes pH 7.4. PK was added at a concentration of 0.2 mg/ml in the presence or absence of 1% triton X-100.

### Endoglycosidase H treatment

Intermediates inserted into isolated DPMs were denatured in glycoprotein denaturing buffer (0.5% SDS and 40 mM DTT) for 30 min at 94 °C. The reaction was incubated at 4 °C for 5 min before the addition of 1x Glyco buffer (50 mM NaOAc pH 6), 5 μL H_2_O and 5000 units of Endo H enzyme, which was then incubated at 37 °C for 30 min. Samples were heated in 2 X sample buffer at 95 °C for 5 min for analysis through Tricine SDS-PAGE.

### Sample analysis

Samples were heated in 2 X sample buffer (4% SDS, 20% Glycerol, 0.12 M Tris pH 6.8, and 10% BME) at 95 °C for 5 min for analysis through Tricine or Tris/Bis SDS-PAGE. The gels were fixed and dried before being exposed to Kodak X-AR film for visualization, and were developed using the X-omat 2000.

## Electronic supplementary material

ESM 1 (DOCX 15 kb)

Figure S1 **TM1 of Bacterioopsin exists in an extended conformation in the ribosome tunnel.** (**a**) The first 65 residues of the prokaryotic protein Bacterioopsin (Bop) are shown, with the position of the single cysteine (C15) residue, required for pegylation, labelled as amino acid 1. TM domains 1 and 2 are highlighted using boldface. Intermediate lengths of Bop, used for in vitro pegylation experiments, are underlined and the length is denoted below in amino acids (aa). Autoradiographs of radiolabeled translation products generated from stalled intermediates of between 25 and 50 amino acids in length were expressed in the (**b**) prokaryotic S-30 coupled transcription/translation system and the (**c**) eukaryotic Wheat Germ (WG) translation system. Intermediate length was measured from the peptidyl-transferase centre (PTC) to the marker cysteine (C15). Translation reactions were split into two, with one half being incubated with 1 mM PEG-MAL (+PEG-MAL) and the other half incubated in buffer as a control (-PEG-MAL). A representative gel displays how the non-pegylated (0) and pegylated (1) samples were resolved by SDS-PAGE (15% Tricine). A gel-shift of ~ 5 kDa occurs if the translation product was successfully pegylated. (**d**) Quantification of the total pegylation of individual intermediates was carried out for both the S-30 transcription/translation system (●) and the WG translation system (▼). The y-axis shows percentage of intermediates successfully pegylated and was obtained by pixel densitometry (Image-J) and calculated using [pegylated band/ (unpegylated band + pegylated band)]. The average percentage pegylation is calculated from an *n* = 3 replicates, error bars are + SD. (PNG 1049 kb)

Figure S2 **F**_**0**_**c, compacts in the ribosome.** F_0_c, is a bacterial membrane protein, specifically subunit c of the Fo component of the ATP synthase. (**a**) The full 79 amino acid sequence of the F_0_c protein is shown with the position of the single cysteine (C5) residue, required for pegylation, labelled as amino acid 1. TM domains 1 and 2 are highlighted using boldface. Intermediate lengths of F_0_c, used for in vitro pegylation experiments, are underlined and the length is denoted below in amino acids (aa). (**b**) The first TM domain of the F_0_c protein was shown to compact by Robinson et al. (2012). Autoradiographs of radiolabeled translation products generated from stalled intermediates of between 35 and 70 amino acids in length were expressed in the prokaryotic S-30 coupled transcription/translation system. Intermediate length was measured from the peptidyl-transferase centre (PTC) to the marker cysteine (C5). Translation reactions were split into two, with one half being incubated with 1 mM PEG-MAL (+PEG-MAL) and the other half incubated in buffer as a control (-PEG-MAL). A representative gel displays how the non-pegylated (0) and pegylated (1) samples were resolved by SDS-PAGE (15% Tricine). A gel-shift of ~ 5 kDa occurs if the translation product was successfully pegylated. Our results agreed with the data presented by Robinson et al. (2012), showing compaction iof TM 1 in the ribosome tunnel. (**c**) Quantification of the total pegylation in the S-30 transcription/translation system, for all intermediates. The y-axis shows percentage of intermediates successfully pegylated and was obtained by pixel densitometry (Image-J) and calculated using [pegylated band/ (unpegylated band + pegylated band)]. The average percentage pegylation is calculated from an n = 3 replicates, error bars are + SD. (PNG 917 kb)

## Abbreviations

*IMPs*: Integral membrane proteins
*TM*: transmembrane
*PTC*: peptidyl transferase centre
*SRP*: signal recognition particle
*ER*: endoplasmic reticulum
*aa*: amino acid
*MC*: marker cysteine
*PEG-MAL*: methoxypolyethylene glycol maleimide
*MK*: marker lysine
*RRL*: rabbit reticulocyte lysate
*WT*: wild type
*NT*: N-terminal
*CT*: C-terminal
*OST*: oligosacchary-ltransferase
*DPMs*: dog pancreas microsomes
*Endo H*: Endoglycosidase H
