# Supplementary material for: Hydrophobicity, rather than secondary structure, is essential for the SRP dependent targeting of GPR35 to the ER membrane
Source: J Bioenerg Biomembr. 2019 Jan 31;51(2):137–50. doi: 10.1007/s10863-019-9785-0 (PMC6439181; doi:10.1007/s10863-019-9785-0)
Supplement: Supplementary file 1 — (DOCX 15 kb) [file 10863_2019_9785_MOESM1_ESM.docx]

| **Plasmid** | **Relevant genotype** | **Source** |
| --- | --- | --- |
| pTrc99a |  | Commercial |
| pcDNA 3.1 |  | Commercial |
| pGPR35 | pTrc99a encoding human GPR35 | This study |
| pcGPR35 | pcDNA 3.1 encoding human GPR35 | This study |
| pGPR35C | pGPR35, C8A and A15C | This study |
| pcGPR35C | pcGPR35, C8A and A15C | This study |
| pGPR35ΔNT | pGPR35, L27E and L31E | This study |
| pGPR35ΔCT | pGPR35, L34E and L40E | This study |
| pcGPR35Δ4E | pGPR35, L27E, L31E, L34E and L40E | This study |
| pcGPR35ΔNT | pcGPR35, L27E and L31E | This study |
| pcGPR35ΔCT | pcGPR35, L34E and L40E | This study |
| pcGPR35Δ4E | pcGPR35, L27E, L31E, L34E and L40E | This study |
| pcGPR35NS_7_T | pcGPR35, Ser insert at pos. 7 | This study |

**Table S1: Plasmids used in this study**

| **Primer name** | **Sequence (5’-3’)** |
| --- | --- |
| pTrc99a for | CTGAAATGAGCTGTTGACAATTAATCATCCGG |
| pcDNA3.1 for | GCAGAGCTCTCTGGCTAACTAGAGAACCCAC |
| GPR35 25aa rev | CAGGCTGTTGAGCAGCAGG |
| GPR35 30aa rev | GAACACCCAGAGCGCCAGGC |
| GPR35 35aa rev | CACTGCTGCATGCGGCAGC |
| GPR35 40aa rev | ATGCGGGTCTCCGTCCAC |
| GPR35 45aa rev | AGGTTGGTCATGTAGATGC |
| GPR35 45aa rev | AGGTTGGTCATGTAGATGC |
| GPR35 50aa rev | AGGTCGGCCACCGCCAGGTTGG |
| SRP GPR35 25aa rev | CATCATCACCCAGAGCGC |
| SRP GPR35 35aa rev | CATCATCTCCGTCCACTG |
| SRP GPR35 45aa rev | CATCATCACCGCCAGGTTGG |
| SRP GPR35 55aa rev | CATCATGGGCAAGGTGCAC |
| SRP GPR35 65aa rev | CATCATTGAGGTGTCTCG |
| Full-length GPR35 rev | TTAGGCGAGGGTCACGCAC |
| GPR35 182aa rev | AACAGCACCCGGCACAATTCATACACTGATACTAGATCTGATAACAGCACCTTATGTCAA |
| 65aa NT Gly Rev | CGACAGCAGGCAGAGGTC |
| 70aa NT Gly Rev | GAAGGGCAAGGTGCACAG |
| 75aa NT Gly Rev | CAGGGAGTGCAGCACGAA |
| 80aa NT Gly Rev | GTCTGAGGTGTCTCGCAG |
| 90aa NT Gly Rev | GATGCCCTGGGAGAGCTG |
| 100aa NT Gly Rev | GCTGATGCTCATGTACC |

**Table S2: Primers used in this study**
