# Supplementary figures and images for: Hydrophobicity, rather than secondary structure, is essential for the SRP dependent targeting of GPR35 to the ER membrane
Source: J Bioenerg Biomembr. 2019 Jan 31;51(2):137–50. doi: 10.1007/s10863-019-9785-0 (PMC6439181; doi:10.1007/s10863-019-9785-0)

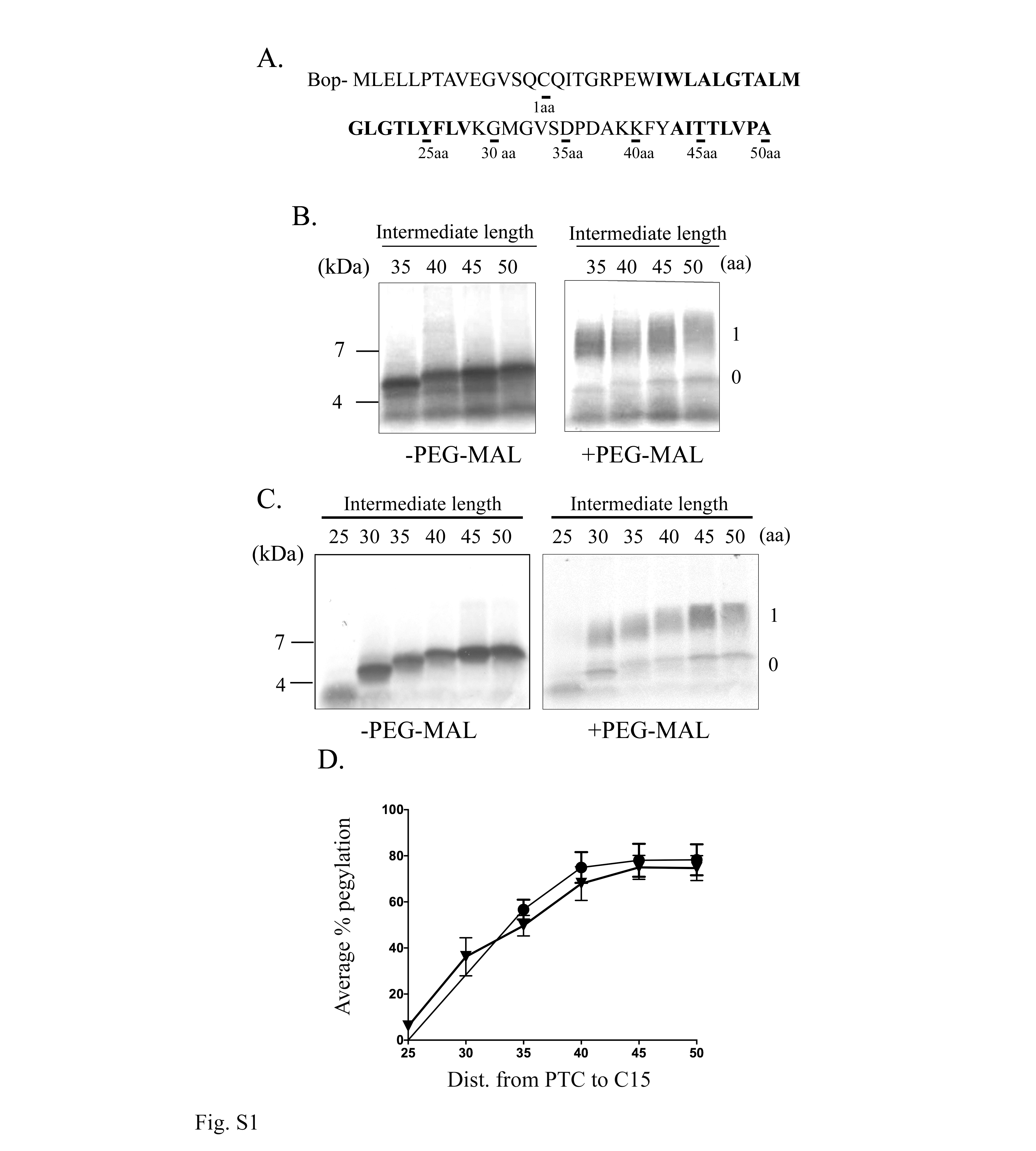

Supplement: Supplementary file 2 — TM1 of Bacterioopsin exists in an extended conformation in the ribosome tunnel. (a) The first 65 residues of the prokaryotic protein Bacterioopsin (Bop) are shown, with the position of the single cysteine (C15) residue, required for pegylation, labelled as amino acid 1. TM domains 1 and 2 are highlighted using boldface. Intermediate lengths of Bop, used for in vitro pegylation experiments, are underlined and the length is denoted below in amino acids (aa). Autoradiographs of radiolabeled translation products generated from stalled intermediates of between 25 and 50 amino acids in length were expressed in the (b) prokaryotic S-30 coupled transcription/translation system and the (c) eukaryotic Wheat Germ (WG) translation system. Intermediate length was measured from the peptidyl-transferase centre (PTC) to the marker cysteine (C15). Translation reactions were split into two, with one half being incubated with 1 mM PEG-MAL (+PEG-MAL) and the other half incubated in buffer as a control (-PEG-MAL). A representative gel displays how the non-pegylated (0) and pegylated (1) samples were resolved by SDS-PAGE (15% Tricine). A gel-shift of ~ 5 kDa occurs if the translation product was successfully pegylated. (d) Quantification of the total pegylation of individual intermediates was carried out for both the S-30 transcription/translation system (●) and the WG translation system (▼). The y-axis shows percentage of intermediates successfully pegylated and was obtained by pixel densitometry (Image-J) and calculated using [pegylated band/ (unpegylated band + pegylated band)]. The average percentage pegylation is calculated from an n = 3 replicates, error bars are + SD. (PNG 1049 kb) [file 10863_2019_9785_MOESM2_ESM.png]

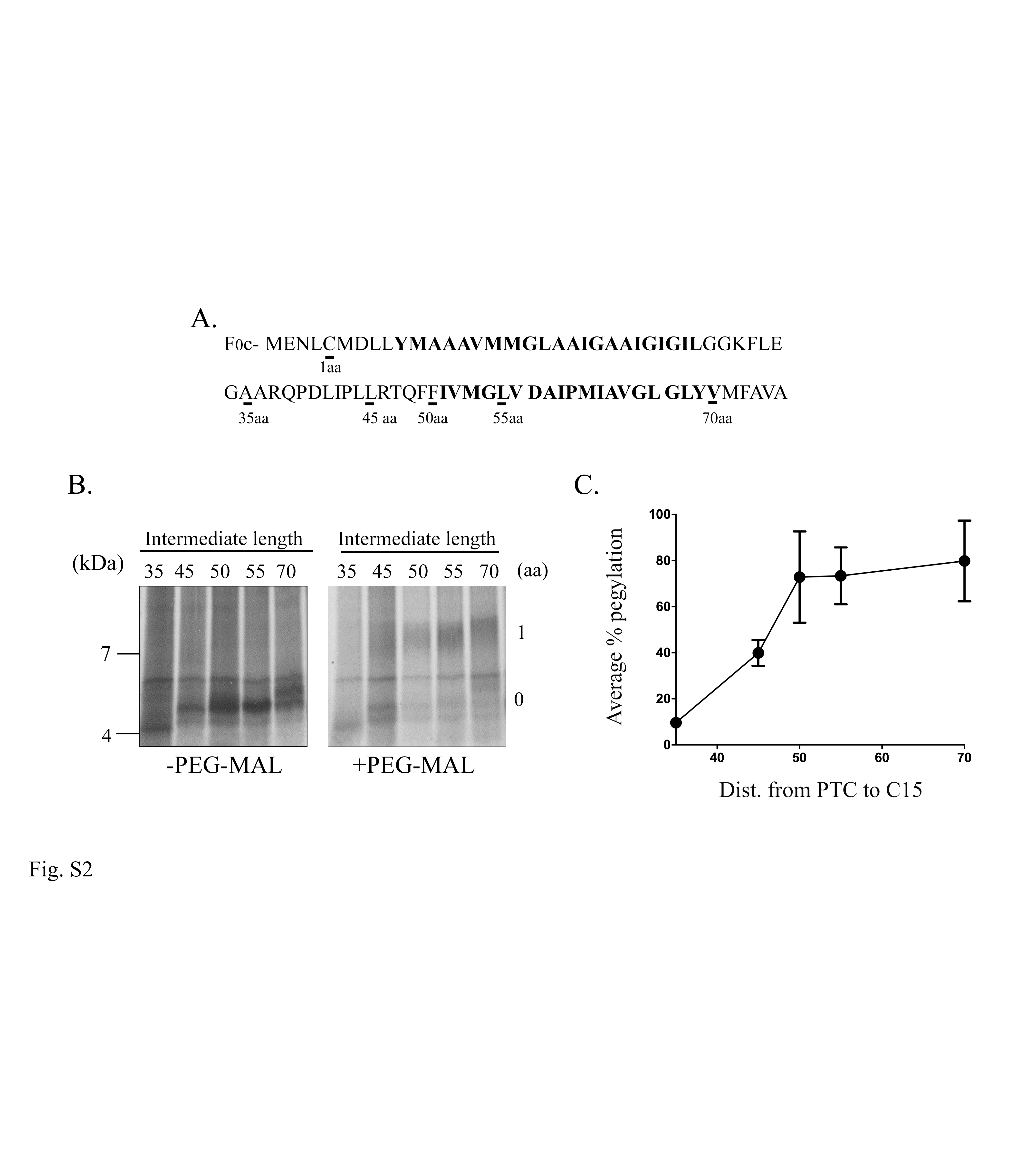

Supplement: Supplementary file 3 — F0c, compacts in the ribosome. F0c, is a bacterial membrane protein, specifically subunit c of the Fo component of the ATP synthase. (a) The full 79 amino acid sequence of the F0c protein is shown with the position of the single cysteine (C5) residue, required for pegylation, labelled as amino acid 1. TM domains 1 and 2 are highlighted using boldface. Intermediate lengths of F0c, used for in vitro pegylation experiments, are underlined and the length is denoted below in amino acids (aa). (b) The first TM domain of the F0c protein was shown to compact by Robinson et al. (2012). Autoradiographs of radiolabeled translation products generated from stalled intermediates of between 35 and 70 amino acids in length were expressed in the prokaryotic S-30 coupled transcription/translation system. Intermediate length was measured from the peptidyl-transferase centre (PTC) to the marker cysteine (C5). Translation reactions were split into two, with one half being incubated with 1 mM PEG-MAL (+PEG-MAL) and the other half incubated in buffer as a control (-PEG-MAL). A representative gel displays how the non-pegylated (0) and pegylated (1) samples were resolved by SDS-PAGE (15% Tricine). A gel-shift of ~ 5 kDa occurs if the translation product was successfully pegylated. Our results agreed with the data presented by Robinson et al. (2012), showing compaction iof TM 1 in the ribosome tunnel. (c) Quantification of the total pegylation in the S-30 transcription/translation system, for all intermediates. The y-axis shows percentage of intermediates successfully pegylated and was obtained by pixel densitometry (Image-J) and calculated using [pegylated band/ (unpegylated band + pegylated band)]. The average percentage pegylation is calculated from an n = 3 replicates, error bars are + SD. (PNG 917 kb) [file 10863_2019_9785_MOESM3_ESM.png]
